# An Ethics Checklist for Digital Health Research in Psychiatry: Viewpoint

**DOI:** 10.2196/31146

**Published:** 2022-02-09

**Authors:** Francis X Shen, Benjamin C Silverman, Patrick Monette, Sara Kimble, Scott L Rauch, Justin T Baker

**Affiliations:** 1 Harvard Medical School Boston, MA United States; 2 Law School University of Minnesota Minneapolis, MN United States; 3 Institute for Technology in Psychiatry McLean Hospital Belmont, MA United States

**Keywords:** digital phenotyping, computataional psychiatry, ethics, law, privacy, informed consent

## Abstract

**Background:**

Psychiatry has long needed a better and more scalable way to capture the dynamics of behavior and its disturbances, quantitatively across multiple data channels, at high temporal resolution in real time. By combining 24/7 data—on location, movement, email and text communications, and social media—with brain scans, genetics, genomics, neuropsychological batteries, and clinical interviews, researchers will have an unprecedented amount of objective, individual-level data. Analyzing these data with ever-evolving artificial intelligence could one day include bringing interventions to patients where they are in the real world in a convenient, efficient, effective, and timely way. Yet, the road to this innovative future is fraught with ethical dilemmas as well as ethical, legal, and social implications (ELSI).

**Objective:**

The goal of the Ethics Checklist is to promote careful design and execution of research. It is not meant to mandate particular research designs; indeed, at this early stage and without consensus guidance, there are a range of reasonable choices researchers may make. However, the checklist is meant to make those ethical choices explicit, and to require researchers to give reasons for their decisions related to ELSI issues. The Ethics Checklist is primarily focused on procedural safeguards, such as consulting with experts outside the research group and documenting standard operating procedures for clearly actionable data (eg, expressed suicidality) within written research protocols.

**Methods:**

We explored the ELSI of digital health research in psychiatry, with a particular focus on what we label “deep phenotyping” psychiatric research, which combines the potential for virtually boundless data collection and increasingly sophisticated techniques to analyze those data. We convened an interdisciplinary expert stakeholder workshop in May 2020, and this checklist emerges out of that dialogue.

**Results:**

Consistent with recent ELSI analyses, we find that existing ethical guidance and legal regulations are not sufficient for deep phenotyping research in psychiatry. At present, there are regulatory gaps, inconsistencies across research teams in ethics protocols, and a lack of consensus among institutional review boards on when and how deep phenotyping research should proceed. We thus developed a new instrument, an Ethics Checklist for Digital Health Research in Psychiatry (“the Ethics Checklist”). The Ethics Checklist is composed of 20 key questions, subdivided into 6 interrelated domains: (1) informed consent; (2) equity, diversity, and access; (3) privacy and partnerships; (4) regulation and law; (5) return of results; and (6) duty to warn and duty to report.

**Conclusions:**

Deep phenotyping research offers a vision for vastly more effective care for people with, or at risk for, psychiatric disease. The potential perils en route to realizing this vision are significant; however, and researchers must be willing to address the questions in the Ethics Checklist before embarking on each leg of the journey.

## Introduction

“The deeper you go, the more you know.” This headline captures the tantalizing promise of deeply probing digital health research in psychiatry [1].

Psychiatry has long needed a better and more scalable way to capture the dynamics of behavior and its disturbances, quantitatively across multiple data channels, at high temporal resolution in real time. By combining 24/7 data—on location, movement, email and text communications, and social media—with brain scans, genetics, genomics, neuropsychological batteries, and clinical interviews, researchers will have an unprecedented amount of objective, individual-level data [2,3]. Analyzing these data with ever-evolving artificial intelligence offers the possibility of intervening early with precision and could even prevent the most critical sentinel events [4]. Ideally, this could one day include bringing interventions to patients where they are in the real world, in a convenient, efficient, effective, and timely way [5,6].

Yet, the road to this innovative future is fraught with ethical dilemmas [7-10], and ethical, legal, and social implications (ELSI) on issues such as informed consent and return of results must be reexamined in light of research employing previously unavailable deep and computational characterization of humans based on: (1) real time and cross-sectional behavioral measures; (2) imaging, interviews, and other phenotypic data; (3) genotypic data; and (4) epigenetic and environmental data. The potentially boundless data collection across these streams gives rise to what might be described as “deep biobehavioral typing” or “deep geno/phenotyping.” Given these multiple streams, ethical frameworks for deep biobehavioral typing must integrate the overlapping ethics of genetics, genomics, ethics of brain imaging, ethics of real time digital monitoring, and so forth to critically reexamine well-known ELSI issues. For example, when considering return of results, although it is true that the deeper you go, the more you know, it is unclear when a researcher knows enough to justify sharing data with a clinician, alerting appropriate individuals about potential self-harm, and returning individual research results [11].

Supported by a National Institutes of Health (NIH) Bioethics Administrative Supplement award (NIH 1U01MH116925-01), we have been exploring the ELSI of digital health research in psychiatry, with a particular focus on what we label “deep phenotyping” psychiatric research, which combines the potential for virtually boundless data collection and increasingly sophisticated techniques to analyze that data. We convened an interdisciplinary expert stakeholder workshop in May 2020, and this checklist emerges out of that dialogue. As we use it in this article, the phrase “deep phenotyping” in psychiatric research is meant to describe research that—even if it does not encompass a large number of research subjects—goes deep into the lives of those subjects by collecting many digital and biological data streams (eg, digital data such as text messages, phone screen shots, and GPS location; health data such as heart rate and blood pressure; and clinical evaluations and biological data such as genetics and brain scans).

Consistent with recent ELSI analyses [8,9], the bottom line of our bioethics analysis is that existing ethical guidance and legal regulation are not sufficient for deep phenotyping research in psychiatry. At present, there are regulatory gaps and inconsistencies across research teams in ethics protocols. There is also a lack of consensus among institutional review boards (IRBs) on when and how deep phenotyping research should proceed [12,13]. Efforts are underway to fill these gaps, notably those led by the Connected and Open Research Ethics initiative at the University of California San Diego [9].

Until the field develops more robust consensus guidelines, however, the onus clearly falls on individual research teams to take the lead in shaping the applied ethics of digital health research in psychiatry.

To guide these ethics considerations, we developed a new instrument, an Ethics Checklist for Digital Health Research in Psychiatry (“the Ethics Checklist”). The Ethics Checklist is composed of 20 key questions, subdivided into six interrelated domains: (1) informed consent; (2) equity, diversity, and access; (3) privacy and partnerships; (4) regulation and law; (5) return of results; and (6) duty to warn and duty to report. The questions included in the checklist are presented in Table 1, and Multimedia Appendix 1 provides the Ethics Checklist as a user-friendly instrument for research teams.

The goal of the Ethics Checklist is to promote the careful design and execution of research. It is not meant to mandate particular research designs; indeed, at this early stage of digital phenotyping research and without consensus guidance, there are a range of reasonable choices researchers may make. But the checklist is meant to make those ethical choices explicit, and to require researchers to give reasons for their decisions related to ELSI issues. The Ethics Checklist is primarily focused on procedural safeguards, such as consulting with experts outside the research group and documenting standard operating procedures for clearly actionable data (eg, expressed suicidality) within written research protocols.

**Table 1:** Ethics checklist for digital health research.

Category	Category description	Checklist items	
Informed consent	How can we meaningfully communicate and be transparent about research methods that involve deep, complex, often passive and continuous data collection, machine learning analysis, and interpretation?	1.	Have we appropriately adapted our informed consent procedures to our specific study population, including possible use of surrogate consent?
		2.	Will we provide background education on relevant technologies, such as explaining what social media companies may already be doing with the participant’s data?
		3.	Have we determined what a reasonable person would want to know, and explained in our institutional review board proposal the evidence on which we reached that determination?
Equity, diversity, and access	How will we address concerns that our research might replicate existing, or generate new, biased results or contribute to health inequities in access based on race, ethnicity, gender, sexual orientation, age, or another legally protected class?	4.	Starting at the early conceptualization and research design stages, have we sought input from a diverse community of stakeholders to identify and address potential equity concerns and opportunities to advance justice with our proposed research?
		5.	Has our research plan addressed potential inequities in access, for instance varying levels of access to mobile technology and to health care services?
		6.	Has every member of the research team completed our institution’s recommended trainings around diversity, inclusion, equity, and access?
Privacy and partnerships	How can we design our research to balance an interest in robust data collection, with a potentially competing interest in protecting participant privacy?	7.	Have we consulted with information security experts about exactly where the data will flow, from start to finish?
		8.	Do we have a written policy on data deidentification and participant privacy that is consistent with best practices in psychiatry and neuroscience?
		9.	Have we determined which, if any, third-party vendors will be required to be HIPAA^a^ compliant and sign a Business Associate Agreement?
Regulation and law	Which state, federal, and international law and regulatory guidance must be adhered to in our research?	10.	Have we examined the terms of service, end user license agreements, privacy statements, and HIPAA notices for each of the vendors and software applications involved in our research?
		11.	Have we determined how laws in applicable jurisdictions will treat the data we collect, for instance considering the data to be “sensitive,” “special category,” or “personal health information”?
		12.	Have we ensured compliance with state, federal and international laws governing our research, HIPAA privacy requirements, state data privacy laws, and applicable international privacy laws?
Return of results	By which criteria will we determine if our data analytic models are sufficiently valid and reliable for us to share the individual research results and data with the research participant and the participant’s clinicians?	13.	Have we considered whether our study will generate any “actionable” results, based on established guidelines and how we have defined actionability?
		14.	Have we established with what frequency results will be returned? (eg, should participants have daily, weekly, and monthly access to some subset of their data?)
		15.	Have we clarified the protocols and mechanisms for returning different types of information, (eg, raw data, interpreted data, etc)?
		16.	Do we have a protocol in place for contacting a participant’s clinicians and nonclinical caregivers?
Duty to warn and duty to report	When might our research trigger a legal or ethical duty to report the potential for participant self-harm or harm to others, and what are our protocols for determining whether in individual instances we have such a duty?	17.	Has everyone in our research lab received sufficient training to know when to flag data or results as requiring follow-up review by a supervisor?
		18.	Will our analytic methods allow us to identify the precursors to dangerous or illegal behavior, to oneself or to others, and if so, at which point will we intervene to protect the research participant or a third party?
		19.	Have we updated our lab’s suicidality standard operating procedure to be consistent with the novel data acquisition and analysis techniques we are using in our study?
		20.	Do we have a protocol for responding to legally mandated reporting if our data uncover child pornography, restraining order violations, and so on?

^a^HIPAA: Health Insurance Portability and Accountability Act.

## The Ethics Checklist in Action

Each of the 20 ethics checklist questions are phrased so that they can be answered with a “Yes,” “No,” or “Pending” response. In our view, deep phenotyping research in psychiatry should not proceed until a research team answers “Yes” or “Pending” to each checklist question. To arrive at “Yes” or “Pending” for each question will require research labs to carefully consider a complex interplay of ethical and legal considerations.

It is beyond the scope of this short paper to address all of these complexities, but we offer here several illustrative examples, from each of the 6 key domains, of how the checklist might be applied in practice.

### Informed Consent

The revised Common Rule requires researchers to present participants with information that is “most likely to assist … in understanding the reasons why one might or might not want to participate in the research,” and that is what “a reasonable person would want to have …” (Title 45 of the Code of Federal Regulations, part 46, effective July 2018) [14]. The Ethics Checklist thus requires researchers to address the question: “Have we determined what a reasonable person would want to know, and explained in our IRB proposal the evidence on which we reached that determination?” There are presently no empirical data or standard protocols for determining what information a reasonable person would want to have before agreeing to participate in deep phenotyping research. Privacy scholars and ethicists are increasingly concerned that the rights of “notice, access, and consent regarding the collection, use, and disclosure of personal data” are no longer adequate because “many privacy harms are the result of an aggregation of pieces of data over a period of time by different entities” [15]. Thus, researchers must consider a broad range of possible information to communicate.

We offer here several of these many possibilities. One decision is whether to provide research participants a list of clear, concrete examples of the inferences that can likely be made from participants’ data. For example, the informed consent material could explicitly say, “You should know that, although we will not reveal this information outside the research team, we may be able to identify when you are going to the bathroom or having sex.” Another decision, especially for researchers who are collecting data on participants’ GPS data and social media content, is whether to provide basic background education to make participants more informed on the data collection and data sharing practices already being utilized by the mobile technology and apps they already use regularly. Third, ethics research has identified a need to make informed consent processes more meaningful and valid by improving communication [16,17]. Research teams may consider innovative strategies such as video-based multimedia as part of the consent procedure [18-20]. They may also consider staged informed consent [21,22], dynamic consent to facilitate 2-way communication between researchers and participants [23], or a systemic oversight approach for big data research that provides flexibility for addressing uncertainty in how data will be used [24].

In addition, researchers in psychiatry must address a further question that has long been challenging for the field, “how to ensure meaningful and valid informed consent with participants who have a mental illness?” [25,26]. The question of decisional capacity in psychiatric research has been well researched, with multiple instruments now available [27], but the field will need to revisit the effectiveness of these instruments in the new context of deep biobehavioral research.

### Equity, Diversity, Inclusion, and Access

It is well established that biomedical research generally [28] and psychiatric research specifically struggle to enroll racially and ethnically diverse participants [29]. In the United States, for example, there is a reluctance of African Americans to participate in research given a long history of racism and exploitation [30,31].

The Ethics Checklist proposes that researchers answer the following question: Starting at the early conceptualization and research design stages, have we sought input from a diverse community of stakeholders to identify and address potential equity concerns and opportunities to advance justice with our proposed research? The question emphasizes that equity concerns extend beyond simply developing proportional samples, and that from the start, “[r]esearch relationships must become balanced, reciprocal, and community informed, without centering researcher and institutional priorities” [32]. Community advisory boards, which can be comprised of community and family stakeholders, can be a useful vehicle for facilitating such engagement [33]. Operationalizing “diverse community of stakeholders” will depend on the nature and scope of the research, institutional context, and affected communities. The stakeholder group will necessarily be comprised differently; for instance, whether the focus is on a single disease versus basic research, or the research involves multiple international sites versus a single community partner.

In defining the stakeholders, the checklist encourages researchers to go beyond their own research team to seek guidance and build trust, even at the conceptual and research design stage. We agree with Wilkins [34], who suggests that enhancing trust “must build on the principles of community engagement including balancing power dynamics, equitable distribution of resources, effective bidirectional communication, shared decision-making, and valuing of different resources and assets (such as the lived experience and knowledge of group norms and perspectives).” Researchers might, for instance, consult with their institution’s leadership on diversity and inclusion to see if the institution already has mechanisms in place for community engagement. Additional options include reviewing the best practices in community-based participatory research and community-engaged research [35] and consulting expertise in other disciplines, including law [36] and the humanities [37].

### Privacy and Partnerships

In an analysis of smartphone digital phenotyping, Onnela and Rauch [38] write that “[p]atient and participant privacy is always of utmost importance in clinical and research settings.” The literature on ethics of deep phenotyping has similarly identified privacy as foundational [39,40]. Deep phenotyping research requires that data flow across multiple platforms and vendors; thus, to safeguard data privacy, researchers must be aware of where the data go, what happens to the data at each stop, and where security vulnerabilities may exist [10]. The Ethics Checklist thus includes the question, “Have we consulted with information security experts about exactly where the data will flow, from start to finish?” Such consultation could potentially lead to modifications in data collection and data analysis techniques to improve privacy safeguards. For instance, security experts may be aware of the vulnerabilities of particular apps or new technical advances recently developed.

### Regulation and Law

The regulation of mobile health apps is currently undergoing transformation [41,42], as is the regulation of artificial intelligence and machine learning data analysis [43]. At the same time, state privacy laws are emerging [44], as are international law innovations such as the European Union’s General Data Protection Regulation [45]. These legal developments have implications for the deep phenotyping research we describe in this article [7].

For instance, the data collection may require interfacing with multiple third-party vendors, and it is the responsibility of the research team to examine the terms of service, end user license agreements, privacy statements, and Health Insurance Portability and Accountability Act (HIPAA) notices for each of these vendors and associated software applications (Checklist question 10). This may not be an easy task, as research on mobile health apps suggests that many vendors do not have a privacy policy publicly available [46]. It will also be challenging to determine how applicable laws will treat the data being collected (Checklist question 11). Different laws define categories differently. For example, what is considered “sensitive” data under the California Consumer Privacy Act and California Privacy Rights Act might be different from what is considered “special category” data under the European Union’s General Data Protection Regulation or considered “personal health information” under HIPAA [47]. In setting up the research design, the research team may need to enlist institutional or external expertise to help understand and comply with these statutes.

In addition, when data collection follows the individual across state or international boundaries, and when data flow across those boundaries, the research will be exposed to multiple legal jurisdictions, including emerging state laws governing privacy and research [48]. For example, if data is collected continuously while a research participant living in Boston visits a relative in Chicago, then goes to a meeting in Baltimore, both the Illinois Biometric Privacy Act and the Maryland Confidentiality of Medical Records Act may apply. This may place different requirements on the research team. Similarly, if data gathered from a research participant in Detroit are transferred to a vendor operating in nearby Windsor, Canada’s data privacy laws may now be relevant in a way they would not be for traditional research with subjects and data firmly rooted in the United States. Given this potential for movement of research subjects, researchers should answer the question, “Have we ensured compliance with state, federal and international laws governing our research, HIPAA privacy requirements, state data privacy laws, and applicable international privacy laws?” Reviewing HIPAA compliance is foundational, and the details of such review are beyond the scope of our commentary; however, we emphasize here that, given the geographic mobility of the subjects, HIPAA is not the only applicable privacy regime. Thus, addressing legal and regulatory concerns may likely require consultation with legal experts in the researcher’s institution. This review of privacy law can be integrated with the Ethics Checklist question 10 concerning vendors’ policies and question 11 concerning the designation of sensitive information.

### Return of Results

The return of individual research results has garnered significant attention in the ethics literature [49-51]. In the context of deep phenotyping research, the return of results is challenging because there are potentially so much data to return and because some of that data fluctuate over time and could be returned hourly, daily, weekly, monthly, and so on. In a virtual workshop we hosted in May 2020, a group of 25 stakeholders from science, medicine, law, and ethics gathered to explore the issues of return of results in deep phenotyping research. The discussion in that workshop made clear that, at present, there is considerable uncertainty over what constitutes “actionable” information in this space as well as divergent practices among research teams in which information participants can access. The Ethics Checklist includes 4 different questions on return of results, including, “Have we considered whether our study will generate any ‘actionable’ results, based on established guidelines, and how have we defined actionability?” The concept of actionability is debated across multiple fields [50,52], and in the context of deep phenotyping it is not clear where the bounds of actionability are.

For instance, if a research team is measuring step count data and a participant’s step count drops below average in a given week, alerting that participant of the data is actionable in the sense that the participant—informed by these data—may choose to walk substantially more steps next week. But what about more complex results, such as a machine learning algorithm that predicts that the participant has a 72% higher likelihood of experiencing a manic episode in the following year? When has the scientific knowledge base accumulated sufficiently to make such a prediction “actionable”? An even more fundamental question is implicated: for any measurement or prediction, what is the confidence in the measurement, sensitivity, or specificity of the interpretation or prediction, and how should that be shared? The effects of researcher mobile health interventions on participants within research studies are only now being studied [53], and the field is still formulating practices for clinical interventions based on phenotyping data [54].

The potential ethical responsibility and legal duty for reanalysis of data is also of concern. For instance, in genomics research, many genetic variations are classified as “variant of uncertain significance (VUS),” but as knowledge increases, those variations may be reclassified [55]. An ethical and legal question is whether researchers should (or must) revisit previously collected data to determine whether reclassification is warranted, and if so, when and how should they contact those participants from the earlier study [56]. This issue will likely emerge in deep geno/phenotyping research, and it should be proactively anticipated with a policy put into place.

### Duty to Warn and Duty to Report

The duty to warn and the duty to report are well known to psychiatric researchers, but the advanced data collection and data analysis methods of deep phenotyping introduce unique concerns [57]. At present, the field has only begun to develop protocols and thresholds for when the data should trigger a legal or ethical duty to report the potential for participants’ self-harm or harm to others, and further work is needed to address the possibility of false positives, false negatives, and reliable signal detection. For instance, among the 4 questions included in the Ethics Checklist under this heading, we require that researchers address the question, “Have we updated our lab’s suicidality standard operating procedure (SOP) to be consistent with the novel data acquisition and analysis techniques we are using in our study?” Traditionally, suicidality SOPs have relied almost exclusively on the clinical judgment of the psychiatrist or psychologist reviewing individual records and conducting interviews with the participant. The goal of deep phenotyping research, however, is to reduce reliance on this single stream of data, and instead to incorporate many additional real-world data points. The suicidality SOP may need to be modified in recognition of this new paradigm of psychiatric assessment. For instance, if GPS data show that the participant has spent 3 hours at a local bar, then is located on a bridge at 2 AM in the morning, and has sent 20 text messages in the past 5 minutes, is there a way for the research team to have such behavior flagged in real time and should there be a real time intervention in the protocol? The Ethics Checklist requires that researchers consider such situations.

## The Urgent Need for Consensus Guidance

Deep phenotyping research offers a vision for vastly more effective care for people with or at risk of psychiatric disease. The potential perils en route to realizing this vision are significant; however, researchers must be willing to address the questions in the Ethics Checklist before embarking on each leg of the journey.

The illustrative examples discussed above make clear that deep phenotyping researchers have few guideposts and little empirical data with which to address many pressing ethical and legal questions critical for their research. This lack of clarity regarding best practices is understandable for a field that has emerged rapidly, mainly in the past 5 years. But as the field continues to expand, there is a need to fill this gap by developing consensus guidance, informed by quantitative and qualitative bioethics research, as well as community and patient advocate input. This paper has raised more questions than answers, and it did not reach many other avenues of inquiry including considerations for international research and research with children.

To make progress toward consensus guidance, we identify 2 immediate action items. First, ethics should be integrated into the practice of deep phenotyping research (as is already being carried out at centers such as the McLean Institute for Technology in Psychiatry and the Connected and Open Research Ethics initiative at the University of California San Diego Research Center for Optimal Digital Ethics in Health).

Second, professional organizations such as the American Psychiatric Association and the Digital Medicine Society, along with institutions such as the NIH and National Academies, are well positioned to convene an interdisciplinary team to conduct in-depth analysis and produce foundational reports to guide the field.

The deeper you go in deep phenotyping research, the deeper the ethical and legal challenges. But with timely, concerted action, the research community can promote ethically sound and legally compliant digital health research in psychiatry.

## Appendix

Multimedia Appendix 1 Ethics checklist for digital health research in psychiatry and worksheet.

## Abbreviations

ELSI: ethical, legal, and social implications
HIPAA: Health Insurance Portability and Accountability Act
IRB: institutional review board
NIH: National Institutes of Health
SOP: standard operating procedure
