# Supplementary material for: An Ethics Checklist for Digital Health Research in Psychiatry: Viewpoint
Source: J Med Internet Res. 2022 Feb 9;24(2):e31146. doi: 10.2196/31146 (PMC8867294; doi:10.2196/31146)
Supplement: Multimedia Appendix 1 [file jmir_v24i2e31146_app1.docx]

**Ethics Checklist for Digital Health Research in Psychiatry - Worksheet**

*How to use this checklist:* The checklist below is intended to promote careful design and execution of digital health research in psychiatry. It is not meant to mandate particular research designs; indeed, at this early stage and without consensus guidance, there are a range of reasonable choices researchers may make. But the checklist is meant to make those ethical choices explicit, and to require researchers to give reasons for their decisions related to ELSI issues. The Ethics Checklist is primarily focused on procedural safeguards, such as consulting with experts outside the research group and documenting standard operating procedures for clearly actionable data (e.g., expressed suicidality) within written research protocols. The checklist is comprised of 20 Yes/No/Pending questions, sub-divided into six key (and interrelated) domains: (1) informed consent, (2) equity, diversity, inclusion, and access, (3) privacy and partnerships, (4) regulation and law, (5) return of results, and (6) duty to warn and duty to report. **Research should not proceed until the PI can answer Yes or Pending to each question in the checklist.**

Date: _____________________________________

Research Project: ___________________________

Project Leader(s): ___________________________

**INSTRUCTIONS:** Carefully review each question, and consult as needed with the research team and experts beyond the research team. Then answer Yes, No, or Pending to each question. If there are “No” answers, develop and implement a follow-up plan to address those items.

| **Informed Consent:** *How can we meaningfully communicate and be transparent about research methods that involve deep, complex, often passive and continuous data collection, machine-learning analysis, and interpretation?* | **No** | **Yes** | **Pending** |
| --- | --- | --- | --- |
| 1. Have we appropriately adapted our informed consent procedures to our specific study population, including possible use of surrogate consent? | □ | □ | □ |
| 2. Will we provide background education on relevant technologies, such as explaining what social media companies may already be doing with the participant’s data? | □ | □ | □ |
| 3. Have we determined what a reasonable person would want to know, and explained in our IRB proposal the evidence on which we reached that determination? | □ | □ | □ |
|  |  |  |  |
| **Equity, Diversity and Access:** *How will we address concerns that our research might replicate existing, or generate new, biased results or contribute to health inequities in access based on race, ethnicity, gender, sexual orientation, age, or another legally protected class?* | **No** | **Yes** | **Pending** |
| 4. Starting at the early conceptualization and research design stages, have we sought input from a diverse community of stakeholders to identify and address potential equity concerns and opportunities to advance justice with our proposed research? | □ | □ | □ |
| 5. Has our research plan addressed potential inequities in access, for instance varying levels of access to mobile technology and to health care services? | □ | □ | □ |
| 6. Has every member of the research team completed our institution’s recommended trainings around diversity, inclusion, equity, and access? | □ | □ | □ |
|  |  |  |  |
| **Privacy and Partnerships:** *How can we design our research to balance an interest in robust data collection, with a potentially competing interest in protecting participant privacy?* | **No** | **Yes** | **Pending** |
| 7. Have we consulted with information security experts about exactly where the data will flow, from start to finish? | □ | □ | □ |
| 8. Do we have a written policy on data deidentification and participant privacy that is consistent with best practices in psychiatry and neuroscience? | □ | □ | □ |
| 9. Have we determined which, if any, third-party vendors will be required to be HIPAA compliant and sign a Business Associate Agreement (BAA)? | □ | □ | □ |
|  |  |  |  |
| **Regulation and Law:** *Which state, federal, and international law and regulatory guidance must be adhered to in our research?* | **No** | **Yes** | **Pending** |
| 10. Have we examined the terms of service, end user license agreements (EULAs), privacy statements, and HIPAA notices for each of the vendors and software applications involved in our research? | □ | □ | □ |
| 11. Have we determined how our laws in applicable jurisdictions will treat the data we collect, for instance considering the data to be “sensitive,” “special category,” or “personal health information” (PHI)? | □ | □ | □ |
| 12. Have we ensured compliance with state, federal and international laws governing our research, HIPAA privacy requirements, state data privacy laws, and applicable international privacy laws? | □ | □ | □ |
|  |  |  |  |
| **Return of Results:** *By which criteria will we determine if our data analytic models are sufficiently valid and reliable for us to share the individual research results and data with the research participant and/or the participant’s clinician(s)?* | **No** | **Yes** | **Pending** |
| 13. Have we considered whether our study will generate any “actionable” results, based on established guidelines and how we have defined actionability? | □ | □ | □ |
| 14. Have we established with what frequency results will be returned, e.g. should participants have daily, weekly, monthly access to some subset of their data? | □ | □ | □ |
| 15. Have we clarified the protocols and mechanisms for returning different types of information, e.g. raw data, interpreted data, and so on? | □ | □ | □ |
| 16. Do we have a protocol in place for contacting a participant’s clinician(s) and non-clinical caregiver(s)? | □ | □ | □ |
|  |  |  |  |
| **Duty to Warn and Duty to Report:** *When might our research trigger a legal or ethical duty to report the potential for participant self-harm or harm to others, and what are our protocols for determining whether in individual instances we have such a duty?* | **No** | **Yes** | **Pending** |
| 17. Has everyone in our research lab received sufficient training to know when to flag data or results as requiring follow-up review by a supervisor? | □ | □ | □ |
| 18. Will our analytic methods allow us to identify the precursors to dangerous or illegal behavior, to oneself or to others, and if so, at which point will we intervene to protect the research participant or a third party? | □ | □ | □ |
| 19. Have we updated our lab’s standard suicidality standard operating procedure (SOP) to be consistent with the novel data acquisition and analysis techniques we are using in our study? | □ | □ | □ |
| 20. Do we have a protocol for responding to legally mandated reporting if our data uncovers child pornography, restraining order violations, and so on? | □ | □ | □ |

FOLLOW-UP PLAN (If needed to address questions with a “No” answer):

- Action item 1:
- Action item 2:
- Action item 3:
